# Diiodidobis{4-[2-(2-methyl­phen­yl)ethen­yl]pyridine-κ*N*}cadmium

**DOI:** 10.1107/S1600536811037573

**Published:** 2011-09-30

**Authors:** Dong Liu

**Affiliations:** a College of Chemistry and Materials Science, Huaibei Normal University, Huaibei 235000, Anhui, People’s Republic of China

## Abstract

In the title complex, [CdI_2_(C_14_H_13_N)_2_], the Cd atom lies on a twofold rotation axis that relates the I atom and the 4-(2-methyl­styr­yl)pyridine ligand to their counterparts. Therefore the asymmetric unit contains one crystallographically independent half-mol­ecule. The Cd atom adopts a tetra­hedral coordination geometry, coordinated by two I atoms and two N atoms from the symmetry-related 4-(2-methyl­styr­yl)pyridine ligands.

## Related literature

For Cd complexes with similar structures, see: Hu & Englert (2002 ▶); Hu *et al.* (2003 ▶). Park *et al.* (2010 ▶). For Cd—I and Cd—N bond lengths, see: Pickardt & Staub (1999 ▶); Deng *et al.* (2009 ▶); Deiters *et al.* (2006 ▶); Amoedo-Portela *et al.* (2003 ▶).
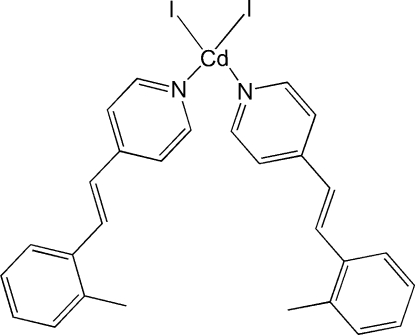


## Experimental

### 

#### Crystal data

[CdI_2_(C_14_H_13_N)_2_]
*M* *_r_* = 756.72
Monoclinic, 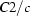

*a* = 26.739 (5) Å
*b* = 7.3613 (15) Å
*c* = 16.072 (3) Å
β = 120.67 (3)°
*V* = 2721.0 (12) Å^3^
*Z* = 4
Mo *K*α radiation
μ = 3.09 mm^−1^
*T* = 223 K
0.35 × 0.30 × 0.25 mm

#### Data collection

Rigaku MercuryCCD area-detector diffractometer
Absorption correction: multi-scan (REQAB; Jacobson, 1998 ▶) *T*_min_ = 0.354, *T*_max_ = 0.452
11814 measured reflections
3106 independent reflections
2204 reflections with *I* > 2σ(*I*)
*R*_int_ = 0.051

#### Refinement

*R*[*F* ^2^ > 2σ(*F* ^2^)] = 0.029
*wR*(*F* ^2^) = 0.058
*S* = 0.83
3106 reflections
151 parameters
H-atom parameters constrained
Δρ_max_ = 0.92 e Å^−3^
Δρ_min_ = −0.57 e Å^−3^

### 

Data collection: *CrystalClear* (Rigaku, 2001) ▶; cell refinement: *CrystalClear* ▶; data reduction: *CrystalStructure* (Rigaku/MSC, 2004 ▶); program(s) used to solve structure: *SHELXTL* (Sheldrick, 2008 ▶); program(s) used to refine structure: *SHELXTL*; molecular graphics: *SHELXTL*; software used to prepare material for publication: *SHELXTL* and *PLATON* (Spek, 2009 ▶).

## Supplementary Material

Crystal structure: contains datablock(s) I, global. DOI: 10.1107/S1600536811037573/bq2302sup1.cif

Structure factors: contains datablock(s) I. DOI: 10.1107/S1600536811037573/bq2302Isup2.hkl

Additional supplementary materials:  crystallographic information; 3D view; checkCIF report

## Figures and Tables

**Fig. 1.**
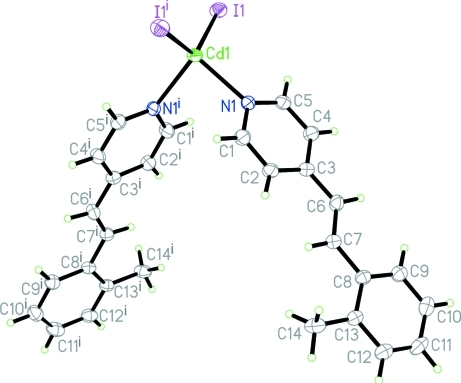
Coordination environment of Cd in the compound with nonhydrogen atoms represented by thermal ellipsoids draw at 30% probability level, hydrogen atoms are drawn as spheres of arbitrary radius. [Symmetry code, i: - x, y, - z - 1/2.]

## supplementary crystallographic information

### Comment

In the past decades, the chemistry of cadmium coordination compounds has attracted much attention owing to their interesting synthetic chemistry and potential applications to luminescence. In this paper, we report the crystal structure of the title compound, a new cadmium complex obtained by the reaction of CdI_2_ and 4-(2-methylstyryl)pyridine.

The title complex crystallizes in the triclinic space group *P*ī, and the asymmetric unit consists of one crystallographically independent half-molecule. As shown in Fig. 1, each Cd atom is tetrahedrally coordinated by two I atoms and two N atoms from two 4-(2-methylstyryl)pyridine ligands. The mean Cd–I and Cd–N bond lengths are similar with those of the reported complexes (Park *et al.*, 2010; Pickardt *et al.*, 1999; Deng *et al.*, 2009; Deiters *et al.*, 2006; Amoedo-Portela *et al.*, 2003).

### Experimental

To a 10 mL Pyrex glass tube was loaded CdI_2_ (37 mg, 0.1 mmol), 4-(2-methylstyryl)pyridine (20 mg, 0.1 mmol) and 3 ml of H_2_O. The tube was sealed and heated in an oven to 160 °C for 3 d, and then cooled to ambient temperature at the rate of 5°C h^-1^ to form yellow crystals.

### Refinement

All the H atoms were placed in geometrically idealized positions (C–H = 0.95 Å for phenyl/pyridyl/vinyl groups and C–H = 0.98 Å for methyl groups) and constrained to ride on their parent atoms with *U*_iso_(H) = 1.2*U*_eq_(C) for phenyl/pyridyl/vinyl groups and *U*_iso_(H) = 1.5*U*_eq_(C) for methyl groups.

### Crystal data

**Table:**

[CdI_2_(C_14_H_13_N)_2_]	*F*(000) = 1448
*M**_r_* = 756.72	*D*_x_ = 1.847 Mg m^−^^3^
Monoclinic, *C*2/*c*	Mo *K*α radiation, λ = 0.71073 Å
Hall symbol: -C 2yc	Cell parameters from 6049 reflections
*a* = 26.739 (5) Å	θ = 3.1–27.5°
*b* = 7.3613 (15) Å	µ = 3.09 mm^−^^1^
*c* = 16.072 (3) Å	*T* = 223 K
β = 120.67 (3)°	Block, yellow
*V* = 2721.0 (12) Å^3^	0.35 × 0.30 × 0.25 mm
*Z* = 4	

### Data collection

**Table:**

Rigaku MercuryCCD area-detector diffractometer	3106 independent reflections
Radiation source: fine-focus sealed tube	2204 reflections with *I* > 2σ(*I*)
graphite	*R*_int_ = 0.051
ω scans	θ_max_ = 27.5°, θ_min_ = 3.3°
Absorption correction: multi-scan (REQAB; Jacobson, 1998)	*h* = −33→31
*T*_min_ = 0.354, *T*_max_ = 0.452	*k* = −9→6
11814 measured reflections	*l* = −20→20

### Refinement

**Table:**

Refinement on *F*^2^	Primary atom site location: structure-invariant direct methods
Least-squares matrix: full	Secondary atom site location: difference Fourier map
*R*[*F*^2^ > 2σ(*F*^2^)] = 0.029	Hydrogen site location: inferred from neighbouring sites
*wR*(*F*^2^) = 0.058	H-atom parameters constrained
*S* = 0.83	*w* = 1/[σ^2^(*F*_o_^2^) + (0.0229*P*)^2^] where *P* = (*F*_o_^2^ + 2*F*_c_^2^)/3
3106 reflections	(Δ/σ)_max_ = 0.001
151 parameters	Δρ_max_ = 0.92 e Å^−^^3^
0 restraints	Δρ_min_ = −0.57 e Å^−^^3^

### Special details

**Table:**

Geometry. All e.s.d.'s (except the e.s.d. in the dihedral angle between two l.s. planes) are estimated using the full covariance matrix. The cell e.s.d.'s are taken into account individually in the estimation of e.s.d.'s in distances, angles and torsion angles; correlations between e.s.d.'s in cell parameters are only used when they are defined by crystal symmetry. An approximate (isotropic) treatment of cell e.s.d.'s is used for estimating e.s.d.'s involving l.s. planes.
Refinement. Refinement of *F*^2^ against ALL reflections. The weighted *R*-factor *wR* and goodness of fit *S* are based on *F*^2^, conventional *R*-factors *R* are based on *F*, with *F* set to zero for negative *F*^2^. The threshold expression of *F*^2^ > σ(*F*^2^) is used only for calculating *R*-factors(gt) *etc*. and is not relevant to the choice of reflections for refinement. *R*-factors based on *F*^2^ are statistically about twice as large as those based on *F*, and *R*- factors based on ALL data will be even larger.

### Fractional atomic coordinates and isotropic or equivalent isotropic displacement parameters (Å^2^)

**Table:**

	*x*	*y*	*z*	*U*_iso_*/*U*_eq_	
Cd1	0.0000	1.22721 (5)	0.7500	0.03840 (11)	
I1	0.053615 (12)	1.38570 (3)	0.66568 (2)	0.05147 (10)	
N1	0.06294 (12)	1.0045 (4)	0.8449 (2)	0.0370 (7)	
C1	0.04379 (17)	0.8800 (5)	0.8835 (3)	0.0472 (10)	
H1	0.0067	0.8965	0.8753	0.057*	
C2	0.07507 (16)	0.7311 (5)	0.9338 (3)	0.0427 (9)	
H2	0.0594	0.6495	0.9595	0.051*	
C3	0.12992 (16)	0.6994 (4)	0.9473 (3)	0.0387 (8)	
C4	0.14992 (16)	0.8297 (5)	0.9084 (3)	0.0462 (10)	
H4	0.1870	0.8169	0.9161	0.055*	
C5	0.11640 (16)	0.9759 (5)	0.8594 (3)	0.0447 (9)	
H5	0.1315	1.0611	0.8345	0.054*	
C6	0.16459 (16)	0.5436 (5)	0.9986 (3)	0.0442 (9)	
H6	0.2028	0.5398	1.0102	0.053*	
C7	0.14763 (16)	0.4041 (4)	1.0314 (3)	0.0386 (8)	
H7	0.1096	0.4109	1.0206	0.046*	
C8	0.18089 (16)	0.2417 (4)	1.0822 (2)	0.0383 (8)	
C9	0.23779 (17)	0.2147 (5)	1.1024 (3)	0.0468 (9)	
H9	0.2551	0.3034	1.0831	0.056*	
C10	0.26921 (18)	0.0622 (6)	1.1497 (3)	0.0553 (11)	
H10	0.3074	0.0467	1.1626	0.066*	
C11	0.2436 (2)	−0.0677 (5)	1.1780 (3)	0.0577 (11)	
H11	0.2644	−0.1731	1.2100	0.069*	
C12	0.18815 (18)	−0.0439 (5)	1.1597 (3)	0.0491 (10)	
H12	0.1716	−0.1337	1.1798	0.059*	
C13	0.15574 (16)	0.1084 (4)	1.1126 (3)	0.0386 (8)	
C14	0.09544 (18)	0.1297 (5)	1.0972 (3)	0.0544 (11)	
H14A	0.0833	0.0160	1.1121	0.082*	
H14B	0.0685	0.1622	1.0304	0.082*	
H14C	0.0957	0.2244	1.1394	0.082*	

### Atomic displacement parameters (Å^2^)

**Table:**

	*U*^11^	*U*^22^	*U*^33^	*U*^12^	*U*^13^	*U*^23^
Cd1	0.0410 (2)	0.03176 (19)	0.0471 (3)	0.000	0.0258 (2)	0.000
I1	0.05478 (19)	0.04516 (15)	0.0666 (2)	0.00138 (12)	0.03979 (17)	0.01120 (12)
N1	0.0339 (17)	0.0353 (16)	0.0432 (18)	−0.0012 (13)	0.0207 (16)	0.0020 (13)
C1	0.042 (2)	0.048 (2)	0.063 (3)	0.0021 (18)	0.035 (2)	0.0091 (19)
C2	0.044 (2)	0.040 (2)	0.053 (3)	−0.0016 (17)	0.031 (2)	0.0103 (18)
C3	0.045 (2)	0.0332 (18)	0.041 (2)	−0.0024 (16)	0.025 (2)	−0.0014 (16)
C4	0.039 (2)	0.043 (2)	0.066 (3)	0.0038 (17)	0.033 (2)	0.0090 (19)
C5	0.045 (2)	0.041 (2)	0.054 (3)	−0.0046 (18)	0.029 (2)	0.0058 (18)
C6	0.034 (2)	0.052 (2)	0.046 (2)	0.0045 (17)	0.019 (2)	0.0080 (18)
C7	0.040 (2)	0.0359 (19)	0.040 (2)	−0.0001 (16)	0.021 (2)	0.0009 (16)
C8	0.047 (2)	0.0330 (18)	0.037 (2)	0.0022 (16)	0.023 (2)	−0.0013 (15)
C9	0.049 (3)	0.046 (2)	0.045 (3)	0.0011 (19)	0.023 (2)	0.0017 (18)
C10	0.048 (3)	0.063 (3)	0.046 (3)	0.012 (2)	0.018 (2)	0.000 (2)
C11	0.071 (3)	0.049 (2)	0.043 (3)	0.019 (2)	0.022 (3)	0.0122 (19)
C12	0.063 (3)	0.040 (2)	0.044 (2)	0.0043 (19)	0.028 (2)	0.0044 (18)
C13	0.049 (2)	0.0348 (18)	0.032 (2)	0.0034 (17)	0.021 (2)	−0.0007 (15)
C14	0.068 (3)	0.040 (2)	0.073 (3)	0.0037 (19)	0.049 (3)	0.0068 (19)

### Geometric parameters (Å, °)

**Table:**

Cd1—N1^i^	2.286 (3)	C7—C8	1.464 (5)
Cd1—N1	2.286 (3)	C7—H7	0.9400
Cd1—I1^i^	2.6898 (5)	C8—C9	1.398 (5)
Cd1—I1	2.6898 (5)	C8—C13	1.410 (4)
N1—C5	1.342 (4)	C9—C10	1.376 (5)
N1—C1	1.346 (4)	C9—H9	0.9400
C1—C2	1.366 (5)	C10—C11	1.380 (5)
C1—H1	0.9400	C10—H10	0.9400
C2—C3	1.389 (4)	C11—C12	1.369 (5)
C2—H2	0.9400	C11—H11	0.9400
C3—C4	1.391 (4)	C12—C13	1.383 (5)
C3—C6	1.441 (5)	C12—H12	0.9400
C4—C5	1.365 (5)	C13—C14	1.510 (5)
C4—H4	0.9400	C14—H14A	0.9700
C5—H5	0.9400	C14—H14B	0.9700
C6—C7	1.335 (4)	C14—H14C	0.9700
C6—H6	0.9400		
			
N1^i^—Cd1—N1	88.38 (14)	C6—C7—C8	128.0 (3)
N1^i^—Cd1—I1^i^	104.30 (6)	C6—C7—H7	116.0
N1—Cd1—I1^i^	112.03 (6)	C8—C7—H7	116.0
N1^i^—Cd1—I1	112.03 (6)	C9—C8—C13	118.3 (3)
N1—Cd1—I1	104.29 (6)	C9—C8—C7	121.7 (3)
I1^i^—Cd1—I1	128.59 (2)	C13—C8—C7	120.0 (3)
C5—N1—C1	115.8 (3)	C10—C9—C8	122.0 (3)
C5—N1—Cd1	125.8 (2)	C10—C9—H9	119.0
C1—N1—Cd1	118.2 (2)	C8—C9—H9	119.0
N1—C1—C2	123.9 (3)	C9—C10—C11	118.8 (4)
N1—C1—H1	118.1	C9—C10—H10	120.6
C2—C1—H1	118.1	C11—C10—H10	120.6
C1—C2—C3	120.4 (3)	C12—C11—C10	120.3 (4)
C1—C2—H2	119.8	C12—C11—H11	119.8
C3—C2—H2	119.8	C10—C11—H11	119.8
C2—C3—C4	115.5 (3)	C11—C12—C13	122.0 (3)
C2—C3—C6	122.9 (3)	C11—C12—H12	119.0
C4—C3—C6	121.6 (3)	C13—C12—H12	119.0
C5—C4—C3	120.9 (3)	C12—C13—C8	118.5 (3)
C5—C4—H4	119.6	C12—C13—C14	119.5 (3)
C3—C4—H4	119.6	C8—C13—C14	122.0 (3)
N1—C5—C4	123.5 (3)	C13—C14—H14A	109.5
N1—C5—H5	118.3	C13—C14—H14B	109.5
C4—C5—H5	118.3	H14A—C14—H14B	109.5
C7—C6—C3	126.2 (3)	C13—C14—H14C	109.5
C7—C6—H6	116.9	H14A—C14—H14C	109.5
C3—C6—H6	116.9	H14B—C14—H14C	109.5

Symmetry codes: (i) −*x*, *y*, −*z*+3/2.
